# Reverse vaccinology and subtractive genomics approaches for identifying common therapeutics against *Mycobacterium leprae* and *Mycobacterium lepromatosis*

**DOI:** 10.1590/1678-9199-JVATITD-2020-0027

**Published:** 2021-04-09

**Authors:** Arun Kumar Jaiswal, Sandeep Tiwari, Syed Babar Jamal, Letícia de Castro Oliveira, Helioswilton Sales-Campos, Leonardo Eurípedes Andrade-Silva, Carlo Jose Freire Oliveira, Preetam Ghosh, Debmalya Barh, Vasco Azevedo, Siomar C. Soares, Virmondes Rodrigues Rodrigues, Marcos Vinicius da Silva

**Affiliations:** 1 Graduate Program in Bioinformatics, Institute of Biological Sciences, Federal University of Minas Gerais (UFMG), Belo Horizonte, MG, Brazil.; 2 Department of Immunology, Microbiology and Parasitology, Institute of Biological Sciences and Natural Sciences, Federal University of Triângulo Mineiro (UFTM), Uberaba, MG, Brazil.; 3 Department of Biological Sciences, National University of Medical Sciences, Rawalpindi, Punjab, Pakistan.; 4 Institute of Tropical Pathology and Public Health, Federal University of Goias (UFG), Goiânia, Goiás, Brazil.; 5 Department of Computer Science, Virginia Commonwealth University, Richmond, VA, USA.; 6 Infectious Disease Department, Institute of Health Sciences, Federal University of Triângulo Mineiro (UFTM), Uberaba, MG, Brazil.; 7 Centre for Genomics and Applied Gene Technology, Institute of Integrative Omics and Applied Biotechnology (IIOAB), Nonakuri, Purba Medinipur, West Bengal, India.

**Keywords:** Mycobacterium leprae, Mycobacterium lepromatosis, Leprosy, Vaccine targets, Drug target identification

## Abstract

**Background:**

*Mycobacterium leprae* and *Mycobacterium lepromatosis* are gram-positive bacterial pathogens and the causative agents of leprosy in humans across the world. The elimination of leprosy cannot be achieved by multidrug therapy alone, and highlights the need for new tools and drugs to prevent the emergence of new resistant strains.

**Methods:**

In this study, our contribution includes the prediction of vaccine targets and new putative drugs against leprosy, using reverse vaccinology and subtractive genomics. Six strains of *Mycobacterium leprae* and *Mycobacterium lepromatosis* (4 and 2 strains, respectively) were used for comparison taking *Mycobacterium leprae* strain TN as the reference genome. Briefly, we used a combined reverse vaccinology and subtractive genomics approach.

**Results:**

As a result, we identified 12 common putative antigenic proteins as vaccine targets and three common drug targets against *Mycobacterium leprae* and *Mycobacterium lepromatosis.* Furthermore*,* the docking analysis using 28 natural compounds with three drug targets was done.

**Conclusions:**

The bis-naphthoquinone compound Diospyrin (CID 308140) obtained from indigenous plant *Diospyros* spp*.* showed the most favored binding affinity against predicted drug targets, which can be a candidate therapeutic target in the future against leprosy.

## Background

Until 2008, the only organism known for causing leprosy was *Mycobacterium leprae*, but then a new species was identified as the causative agent of diffuse lepromatous leprosy (DLL). The newly identified species was *Mycobacterium lepromatosis*, obtained from the blood sample of two patients of Mexican origin who passed away because of the disease and identified as a causative agent for atypical leprosy [1-3]. This disease may occur at any age, mostly affects the skin, peripheral nerves, mucosal surface of the upper respiratory tract and eyes [4, 5]. In terms of microbiology, *M. lepromatosis* is much similar to *M. leprae*, and both species are non-cultivable, acid-fast, and have the ability to infect peripheral nerves. Clinically and microbiologically, these two organisms are so similar that they were counseled to represent the “*M. leprae* complex” Singh *et al.* [6], like the Mycobacterium species that denotes the tuberculosis complex [7]. The transmission mechanism of leprosy is still uncertain; it is hypothesized to be transmitted by the firm contact between leprosy-infected and healthy individuals [8]. Emerging trends however point out to other possibilities of transmission through insects, which cannot be debarred completely [4, 8]. The usual symptoms of the disease are skin lesions, which could be macule (flat), papules (raised) or nodules, and sensory loss [8]. According to WHO, 211,973 new cases of leprosy were reported globally in 2015 (2.9 new cases/ 100,000 people). Global statistics show that 94% of leprosy cases were reported in only fourteen countries and a high number of new cases indicate the degree of unremitting transmission of infections [9, 10]. Approximately, 81% of the new cases worldwide are accounted from Brazil, India and Indonesia where it is currently the most endemic [11]. WHO’s evaluation of Brazilian cases between 2011 and 2013 reveals ten areas with the highest endemicity, which is located mainly in the states of Bahia, Goiás, Mato Grosso, Maranhão, Pará, Rondônia and Tocantins. These places however represent almost 14% of the Brazilian population [11, 12].

None of the laboratory tests is considered sufficient enough to diagnose leprosy. Usually, clinical data accompanied with semiological techniques like evaluation of skin sensitivity and pilocarpine or histamine testing, can conclude the diagnosis [8, 13].

Presently, the diagnosis of leprosy is carried out by expert clinicians using well-defined criteria, beside the use of slit-skin smears and biopsies. With the decrease in occurrence of the disease, clinical expertise is also shrinking, leading to prolonged delays between onset of clinical signs and identification of disease, resulting in improper maintenance of transmission of *M. leprae*. Hence, efforts to eradicate the disease are undermined. In the absence of impeccable tests to detect all *M. leprae* infected individuals, a diagnostic test to confirm leprosy at initial stages among symptomatic patients would be an adequate and certainly useful shorter-term conciliation [14].

Although leprosy is curable, the emergence of antibiotics resistant strains is of major concern and highlights the risk of the disease, especially for those that are under secondary prevention (chemotherapy) as the main component of their control strategy [8, 13]. The multidrug therapy (MDT) was the major factor to decrease the leprosy burden from 1981 until the year 2005; afterwards, slower reduction was reported as Rifampicin resistance in various endemic areas against leprosy, which was the backbone of multidrug therapy of leprosy [9, 15]. Because of this Rifampicin resistance, fluoroquinolones became the preferred category of second-line drugs. Unfortunately, the stains of *M. leprae* with quinolone-resistance have been reported in several countries [16]. This might be because of the wide use of quinolones for treating numerous types of infections. To meet the problem of containing the disease and responding to an increasing circulation of drug-resistant strains, it is essential to assess drug-sensitivity patterns globally [17-19]. These two organisms are microbiologically and clinically very similar but there is a 9% difference at the genome level that was reported in [6]. For the rapid identification of novel vaccine targets, reverse vaccinology is a popular and more conventional approach in the post-genomic era. Approaches like comparative and subtractive genomics and differential genome analyses are extensively used for therapeutic target identification in several human pathogens, including *M. leprae* [10], *M. tuberculosis* [20] *Treponema pallidum* [21], *Haempphilis ducrei* [22], *Mycoplasma pneumonae* [23], *Corynebacterium diphtheriae* [24] and many other pathogenic microorganisms. Here, in this work we applied the integrative *in silico* approaches of reverse vaccinology and subtractive genomics on *M. leprae* and *Mycobacterium lepromatosis* strains to identify common putative therapeutic targets from the genomic information. Furthermore, this study identifies plant-derived lead antimicrobial compounds, with favorable interactions, lowered energy values, and high complementarity with the predicted drug targets.

## Methods

### Data retrieval

The genome sequences of all six strains of *M. leprae* and *M. lepromatosis* (4 and 2 strains, respectively) were retrieved from the NCBI database (https://www.ncbi.nlm.nih.gov/genome/genomes/903&https://www.ncbi.nlm.nih.gov/genome/genomes/36766). All genomes were annotated using the RAST server Rapid Annotation using Subsystem Technology] [25] for the homogenization in the functional annotation. The genome of *M. smegmatis* was used as a non-pathogenic reference where applicable.

### Identification of conserved non-host homologous, pathogenicity islands and genomic islands

We compared 6 strains of *M. leprae* keeping *M. leprae* TN as the reference genome, using orthoMCL software [26], with an E-value of 1e-50. Proteins shared by all strains were considered to be a part of the core genome. To avoid the autoimmunity, identified candidates for drug and/or vaccine should be non-homologues to Human [21, 24, 27]. Therefore, these core genes were subjected to orthoMCL software with default parameter (E-value = 1e-50 and >98% identity over >98% of query sequence length) against human genome for the identification of non-host homolog targets. The identification of pathogenicity islands in the genome of *M. leprae* TN was performed with GIPSy (Genomic Island Prediction Software) [28] through the detection of regions presenting: deviations in genomic signature, i.e., anomalous G+C and/or codon usage deviation; presence of transposase, virulence or flanking tRNA genes; and absence in the non-pathogenic organism *Mycobacterium smegmatis.*

### **Reverse vaccinology approach for the prediction of putative vaccine targets against *M. leprae* and *M. lepromatosis*.**

The non-host homologous conserved proteome of *M. leprae* TN was screened using SurfG+ software [29] to identify secreted, membrane and putative surface exposed proteins. We searched cleavage sites and transmembrane helices and functional domains in the protein to identify vaccine candidates by online tools, SignalP predicts the presence of signal peptides and the location of their cleavage sites in proteins in microorganisms [30], TMHMM (predict the trans-membrane helix in protein) [31] and InterProScan (InterPro provides functional analysis of protein sequences by predicting the presence of domains and important sites as well as classify them into families) [32]. Furthermore, the dataset was screened by Vaxign [33], an online web tool based on reverse vaccinology approach, to search for proteins with the following features: Major histocompatibility complex (MHC) I and MHC II binding properties; adhesion probability greater than 0.51; and no similarity to host proteins.

### High throughput structural modelling and prioritization of identified drug targets

MHOLline (http://www.mholline.lncc.br) was used to predict the 3D models of a complete set of proteins for the whole conserved core of the non-host homologous proteome. MHOLline utilizes multi-fasta file of amino acids as an input data for model generation using the MODELLER program. The adopted methodology was revised accordingly from the original work published earlier [21, 24, 34-36]. The final identified candidate drug targets were prioritized based on criteria (i) the target must have no homology with host; (ii) the target must be a core gene of the pathogen; (iii) the target involved in the pathogen’s unique pathway or multiple pathways are considered superior; (iv) pathways with multiple targets are superior to those having just a single target; (v) in the case of enzyme, targets in host-pathogen common pathways, should not be of the same class of protein, and the EC. no. (Enzyme Commission number) of the target should not match that of any protein product of the host; and (vi) the targets related to pathogenic island or virulence proteins are considered superior, as described by Barh *et al.* [27], Hassan *et al*. [35] and Jamal *et al*. [24] [21, 34-36]. For this, the pathways of identified drug target proteins have been checked using (KEGG Kyoto Encyclopedia of Genes and Genomes) [37], and functionality analysis (Molecular Function and Biological Process) was done using UniProt Universal Protein Resource [38, 39]. Furthermore, PAIDB (Pathogenicity island database) [40] was used to cross check the virulence other than GIPSy [28].

### Ligand library and docking

The ligand library of 28 natural compounds was used for docking from Tiwari *et al*. [41] and Jaiswal *et al*. [21]. The 3D structures of all target proteins were carefully analyzed for structural error in ADT (Auto Dock Tool), and MGLTool (Molecular Graphics Laboratory, version-1.5.4) [42]. Grid box parameters and configuration files were generated separately for all targets. Configuration files for the targets Ml_TN_0449, Ml_TN_1385 and Ml_TN_3807, almost covering the whole proteins, were set as described below. Target Ml_TN_0449 (ML0294- ThiC): Nº of points in *X*-dimension:112, *Y*-dimension:110, *Z*-dimension:126 and Center Grid Box: *X* center:45.273, *Y* center:34.286 and *Z* center:1.003. Target Ml_TN_1385 (ML0808): Nº of points in *X*-dimension:90, *Y*-dimension:98, *Z*-dimension:76 and Center Grid Box: *X* center:22.435, *Y* center:30.15 and *Z* center:26.14. Target Ml_TN_3807 (ML2123): Nº of points in *X*-dimension:98, *Y*-dimension:106, *Z*-dimension:94 and Center Grid Box: *X* center:2.988, *Y* center:20.517 and *Z* center:41.766. The molecular docking was carried out via Autodock vina [43], a program for molecular docking and virtual screening. The Shell and Python scripts vina_screen_local.sh and vina_screen_get_top.py were used for virtual screening and for identifying the top molecule. The 3D poses of docked molecules were analyzed in Chimera [44]. Molecular function (MF) and biological process (BP) for each target protein were determined using UniProt [38, 39]. The biochemical pathways of these proteins were checked using KEGG (Kyoto Encyclopedia of Genes and Genomes) [37] and SurfG+ software [29], and their virulence was checked using GIPSy [28].

## Results

**Figure 1.**
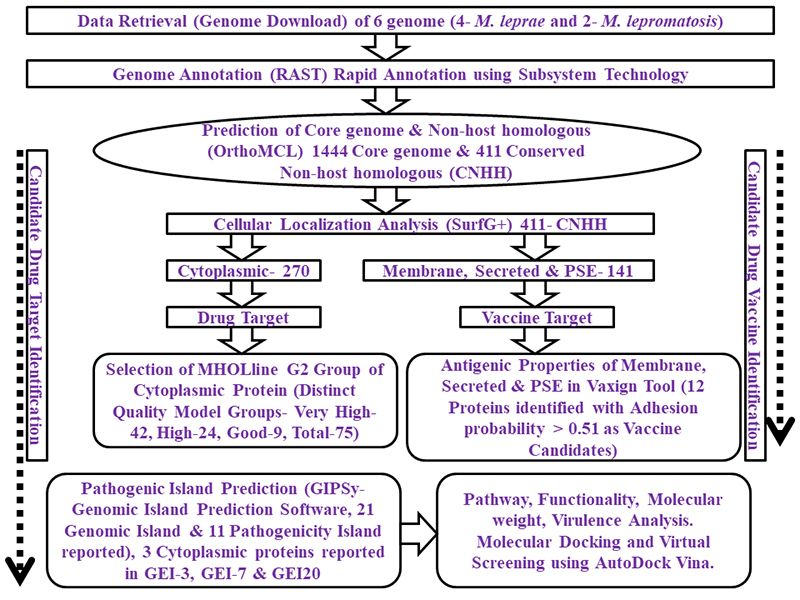
The complete workflow with the methodologies used and the total number of proteins identified in each step.

Total number of proteins described in each section and methodologies used in this work are described in the workflow (Figure 1).

### Identification of conserved non-host homologous proteins and pathogenicity island

**Figure 2.**
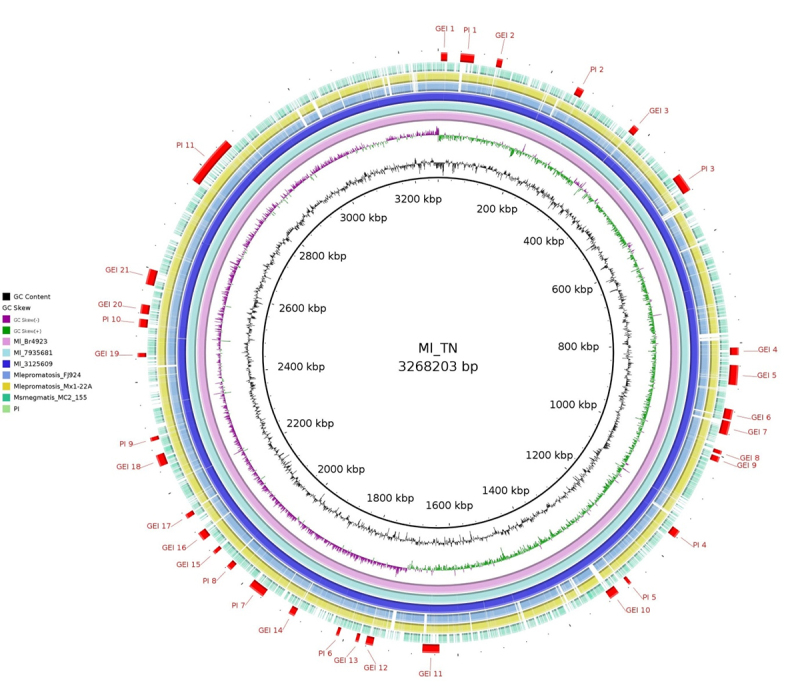
Circular genomic representation of islands (PIs and GIs) in the genomes of *M. leprae* and *M. lepromatosis*. All genomes were aligned using *Mycobacterium leprae* TN strains as reference. The outer-most region highlighted in red shows GIs (21), PI (11) and GC content is shown in black.

Comparative genomics approach was carried out in order to cluster orthologous genes to get a framework to incorporate information from multiple genomes, highlighting the conservation and divergence of gene families and biological processes; for pathogens clustering, orthologs can simplify the identification of drug and/or vaccine targets. We compared 6 strains of *M. leprae* and *M. lepromatosis* (4 and 2 strains, respectively) (Table 1) keeping *M. leprae* TN as reference using the orthoMCL software [26]. A total of 1444 shared proteins by all species were considered to be a part of the core genome. Considering human as a host, a set of 411 conserved non-host homologous proteins were identified. The knowledge about pathogenicity islands, the virulence factors, and their mobility structure is helpful in understanding the bacterial evolution and their interactions with host cells [45]. The prediction of Genomic islands GIs] were subsequently performed using GIPSy. GIs are gene clusters, usually >8 kb in size, likely acquired via horizontal gene transfers (HGT) [28]. GIs considerably influence bacterial evolution and play a role in the environmental or host adaptation of bacterial species [46]. For *M. leprae* and *M. lepromatosis* strains, 32 putative GIs were identified through GIPSy using *M. smegmatis* as a closely related non-pathogenic organism. Of the 32 GIs, 11 are classified as pathogenicity islands (PAIs), i.e., they present high concentration of virulence factors and are absent in the aforementioned closely related non-pathogenic organism (Figure 2).

**Table 1.** Genomic features of *M. leprae* and *M. lepromatosis* strains used in this analysis.

Strain	Size Mb	GC%	Gene	Protein
*M. leprae*_TN	3.26	57.80	2770	1605
*M. leprae*_Br4923	3.26	57.80	2796	2251
*M. leprae*_7935681	3.26	57.80	2842	2303
*M. leprae*_3125609	3.26	57.70	2831	2312
*M. lepromatosis*_FJ924	3.21	58.00	2811	2027
*M. lepromatosis*_Mx1-22A	3.20	57.90	2826	2181

### Localization and vaccine target prediction

**Figure 3.**
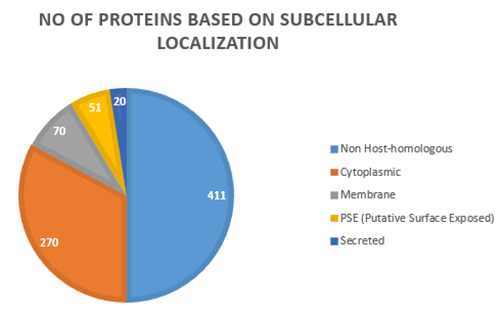
The graphical representation of core non-host homologous proteins by subcellular localization. About 411 non-host homologous proteins were used in localization analysis, 270 proteins belong to the cytoplasmic category, 70 proteins belong to membrane, 51 PSE and 20 proteins identified as secreted proteins.

The possible vaccine target identification, subcellular localization and the secretion of pathogenic proteins are important factors for consideration. The secreted and membrane proteins are the first to be in contact with the host, signaling an immune response. Thus, the prediction of the exo-proteome or secretome, composed of the proteins limited to the extracellular matrix or outer membrane of the organism, is of great importance for reverse vaccinology strategies. Therefore, reverse vaccinology in combination with subtractive genomics can offer more reliable output as compared to screening of the whole dataset without taking into account the prioritizing parameters [27, 47]. The subcellular localization of conserved non-host homologous proteins of *M. leprae* and *M. lepromatosis* strains were predicted with SurfG+. We identified 141 genes as putative surface-exposed (PSE) proteins, secreted proteins or membrane proteins and 270 cytoplasmic proteins (Table 2, Figure 3). We used 141 proteins to predict vaccine candidates with adhesion probability of 0.51 using Vaxign. We identified 12 proteins in *M. leprae* strain TN, which are commonly shared with *M. lepromatosis* and may be considered as potential common vaccine candidates for the leprosy disease*.*

**Table 2.** Number of proteins identified after subcellular location in different categories.

Cytoplasmic protein	270
Membrane protein	
PSE^a^	141
Secreted protein	

^a^ Putative surface exposed

Some prior computational and experimental studies on *M. leprae* have identified antigenic targets for the development of vaccine, where they have shown antigens that are recognized by antibody response of the patients [10, 48, 49]. However, they have only focused on antigenic targets against *M. leprae*. In our reverse vaccinology analysis, we have also worked with *M. lepromatosis* to identify a more global vaccine against all manifestations of the disease (i.e., leprosy and diffuse lepromatous leprosy). We have identified 12 vaccine targets (Table 3) with adhesion probabilities greater than 0.51; interestingly, we found that the protein diacylglycerol acyltransferase/mycolyltransferase (ML0098/ NP_301196.1) was identified as an antigenic protein in the previous *in vitro* study of Kumar *et al*. [21], which may validate the importance of our *in silico* predictions for the identification of common vaccine candidates against the leprosy disease.

**Table 3.** Putative vaccine candidate targets identified by Vaxign.

Locus_tag	Protein ID	Gene name	Subcellular localization	Gene product	NCBI gene product	SignalP result cleavage site	TMHMM	InterProScan domain	Adhesion probability
Ml_TN_1521 ML0877	NP_301663.1	mmpS3	MEM	Putative membrane protein MmpS3	Hypothetical protein ML0877 [*Mycobacterium leprae* TN]	NO	TMH=1	No	0.615
Ml_TN_3055 ML1720	NP_302185.1	-	PSE	Hypothetical protein	Hypothetical protein ML1720 [*Mycobacterium leprae* TN]	Yes Between 19&20	TMH=0	No	0.534
Ml_TN_3545 ML1991	NP_302342.1	-	PSE	PPE family protein	PPE family protein [*Mycobacterium leprae* TN]	NO	TMH=2	No	0.574
Ml_TN_0138 ML0091	NP_301189.1	pirG	SEC	Exported repetitive protein cell surface protein PirG]	Hypothetical protein ML0091 [*Mycobacterium leprae* TN]	Yes Between 22&23	TMH=1	No	0.665
Ml_TN_4191 ML2308	NP_302503.1	pon1	SEC	Multimodular transpeptidase-transglycosylase EC 2.4.1.129]	Penicillin binding protein [*Mycobacterium leprae* TN]	NO	TMH=0	Glycosyl transferase, family 51 IPR001264]; Penicillin-binding protein, transpeptidase IPR001460]; PASTA domain IPR005543]	0.553
Ml_TN_4176 ML2295	NP_302490.1	-	SEC	Protease	Peptidase [*Mycobacterium leprae* 7935681]	Yes Between 20&21	TMH=0	No	0.545
Ml_TN_3237 ML1811	NP_302232.1	-	SEC	Invasion protein - Putative exported p60 protein homologue	Hypothetical protein ML1811 [*Mycobacterium leprae* TN]	Yes Between 31&32	TMH=1	Endopeptidase, NLPC/P60 domain IPR000064]	0.657
Ml_TN_2664 ML1506	NP_302056.1	-	SEC	Hypothetical protein	Hypothetical protein ML1506 [*Mycobacterium leprae* TN]	Yes Between 26&37	TMH=0	No	0.651
Ml_TN_2315 ML1334	NP_301958.1	-	SEC	Possible membrane protein	Hypothetical protein ML1334 [*Mycobacterium leprae* TN]	NO	TMH=1	Domain of unknown function DUF4333 IPR025637]	0.571
Ml_TN_3419 ML1918	NP_302292.1	-	SEC	Hypothetical protein	Hypothetical protein ML1918 [*Mycobacterium leprae* TN]	Yes Between 45&46	TMH=1	No	0.560
Ml_TN_0146 ML0098	NP_301196.1	mpt51	PSE	Antigen 85-C precursor 85C] Antigen 85 complex C] Ag85C] Mycolyl transferase 85C] EC 2.3.1.-]	Diacylglycerol acyltransferase/mycolyltransferase [*Mycobacterium leprae* TN]	NO	TMH=1	No	0.581
Ml_TN_1871 ML1099	NP_301805.1	lprE	PSE	Putative lipoprotein IprE Precursor	Lipoprotein [*Mycobacterium leprae* TN]	Yes Between 50&51	TMH=0	No	0.526

### High throughput structural modelling

Cytoplasmic proteins are also very important for the physiology of bacteria, as they are involved in many important metabolic functions. The pivotal role of cytoplasmic proteins in the maintenance of cell viability makes them more favorable as drug targets [47, 50]. Therefore, the identified 270 cytoplasmic proteins were submitted to the online tool MHOLline for prediction of their 3D structure. The transmembrane regions are detected by the program HMMTOP. The BLAST algorithm was used for the identification of template structures by performing a random search against the Protein Data Bank (PDB) [51]. Blast Automatic Targeting for Structures (BATS) performs the refinement in the template search. BATS selects the best template for 3D model generation and performs automated alignment used by the Modeller program. Moreover, it gathers all the BLAST output files into four distinctive groups, i.e. G0, G1, G2, and G3, according to the following criteria; G0 = Not aligned sequence; G1 = E-value > 10e^-5^ or Identity < 15%; G2 = E-value ≤ 10e^-5^ and Identity ≥ 25% AND LVI ≤ 0.7; G3 = E-value ≤ 10e^-5^ and Identity ≤ 15% and <25% OR LVI> 0.7. Length Variation Index (LVI) is a concept of coverage calculated by the MHOLline software to identify the number of aligned amino acids between query and subject sequences (LVI ≤ 0.1 is equivalent to a coverage ≥ 90%). Only the first three distinct quality model groups of G2 were taken into consideration in this study; these were: 1- Very High quality model sequences (identity ≥ 75%) (LVI ≤ 0.1), 2- High quality model sequences (identity ≥ 50% and < 75%) (LVI ≤ 0.1), and 3- Good quality model sequences (identity ≥ 50%) (LVI > 0.1 and ≤ 0.3) (http://www.mholline.lncc.br) [21, 24, 34]. Therefore, all the considered protein 3D models were constructed from sequences for which the template was available with identity ≥ 50%. We identified 75 proteins (Very High: 42, High: 24 and Good: 9) in the first three distinct quality model groups of G2. After that, out of these 75 proteins, the ones that were present in any identified GIs were reported as candidate drug targets. As a result, we found 3 non-host homologues proteins. Furthermore, these 3 proteins were considered for the drug target prioritization and docking studies (Table 4).

**Table 4.** The identified three drug targets with its functional annotation and prioritization parameters.

Protein ID gene name locus tag	Official name	Mol. Wt^a^ (KDa)	Functions^b^	Cellular component^c^	Pathways^d^	Virulence^e^
**Ml_TN_3807 NP_302402.1 ML2123**	Two-component system response regulator	25.11	**MF:** DNA binding **BP:** phosphorelay signal transduction system, regulation of ,transcription,	Cytoplasm	Unknown	Yes
**Ml_TN_1385 entC ML0808**	Putative phosphoglycerate mutase family protein	20.94	**MF:** isochorismate synthase activity **BP:** biosynthetic process	Cytoplasm	Biosynthesis	Yes
**Ml_TN_0449 NP_301331.1 ThiC ML0294**	Thiamine biosynthesis Protein Thic	59.84	**MF:** Lyase activity, Zinc ion binding **BP:** Thiamine biosynthetic process	Cytoplasm	Thiamine diphosphate biosynthesis	Yes

^a^ Molecular weight was determined using the ProtParam tool (http://web.expasy.org/protparam/).

^b^ Molecular function (MF) and biological process (BP) for each target protein was determined using UniProt.

^c^ Cellular localization of pathogen targets was performed using SurfG+.

^d^ KEGG was used to find the role of these targets in different cellular pathways.

^e^ PAIDB and GIPSy were used to check if the putative targets are involved in pathogen virulence.

### Virtual screening and molecular docking of non-host homologous targets

For each targeted protein (Ml_TN_0449-ML0294 NP_301331.1ThiC), Ml_TN_1385-ML0808 (entC), Ml_TN_3807-ML2123 (NP_302402.1), 28 natural antimicrobial compounds were docked to examine each molecule individually for the selection of the final set of promising molecules that showed favorable interactions with the active residues of the target. We considered the lowest Autodock vina binding affinity for the molecules and interactions with the target residues. The biological importance for each target is described in Table 4 along with an analysis of the predicted protein-ligand interaction(s). The name of molecules, Autodock vina binding affinity scores for the selected ligands and number of predicted hydrogen bonds with the interacting residues involved are shown for each target protein (Tables 5-7).

**Figure 4.**
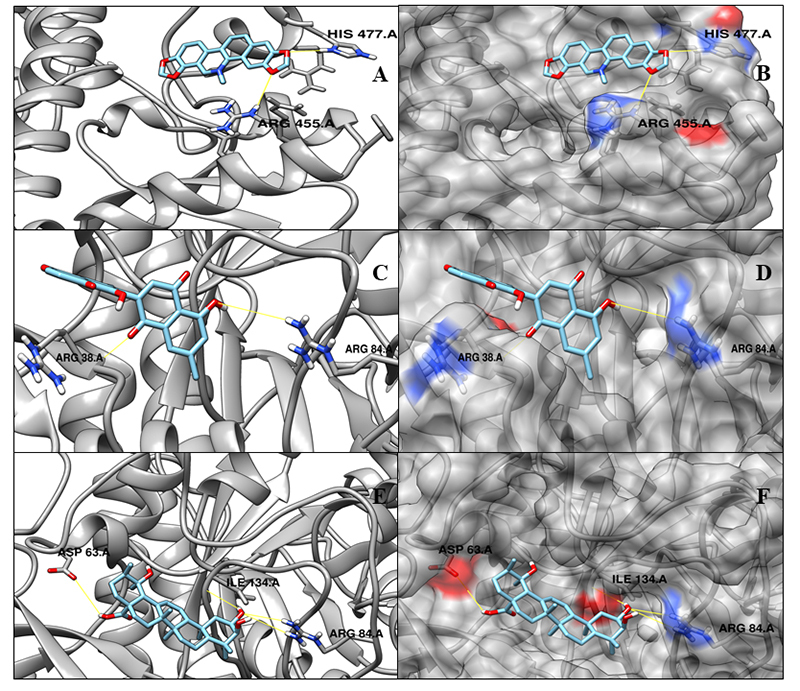
Three-dimensional representation of docking analysis for the target ML0294 (ThiC). **(A)** Cartoon representation with molecule CID 5154 (Sanguinarine). **(B)** Surface representation with molecule CID 5154 (Sanguinarine). **(C)** Cartoon representation with molecule CID 308140 (Diospyrin). **(D)** Surface representation with molecule CID 308140 (Diospyrin). (**E)** Cartoon representation with CID 73645 (Jacarandic Acid). **(F)** Surface representation with CID 73645 (Jacarandic Acid).

NP_301331.1 (ThiC, Thiamine biosynthesis Protein) is the only known enzyme *in vivo* that is required for the conversion of AIR (5-amino-imidazole ribonucleotide) to HMP-P (4-amino-5-hydroxymethyl-2-methylpyrimidine phosphate). Inhibitors of these enzymes are capable of blocking the endogenous Thiamine biosynthesis leading to vitamin deficiency, and hence responsible for damaging the survival and growth of the cell. It has been reported as possible drug target for *M. tuberculosis* [52]. Essential enzymes of the thiamine biosynthetic pathway are possible targets for antibiotic development [53]. Based on the crystallographic structural comparison of the ThiC template (PDB ID: 4S28), none of the active site residues were identified. The docking analysis was performed to identify the minimum energy binding affinity score. Table 5 and Figure 4 show the set of 3 most interacting ligands according to their minimum affinity and the number of hydrogen bond interactions.

**Table 5.** Autodock vina score for the selected best-ranked natural compounds with target ML0294 (ThiC) and predicted hydrogen bonds.

Ml_TN_0449 ML0294 (NP_301331.1, ThiC) - Thiamine biosynthesis protein ThiC		
Compound name	Autodock vina binding affinity	N^o^ of H-bond/residues
CID 5154 (Sanguinarine)	-6.8	3/ARG455, HIS477
CID 308140 (Diospyrin)	-8.6	2/ARG84, ARG38
CID 73645 (Jacarandic Acid)	-8.1	4/ASP63, ILE134, ARG84

**Figure 5.**
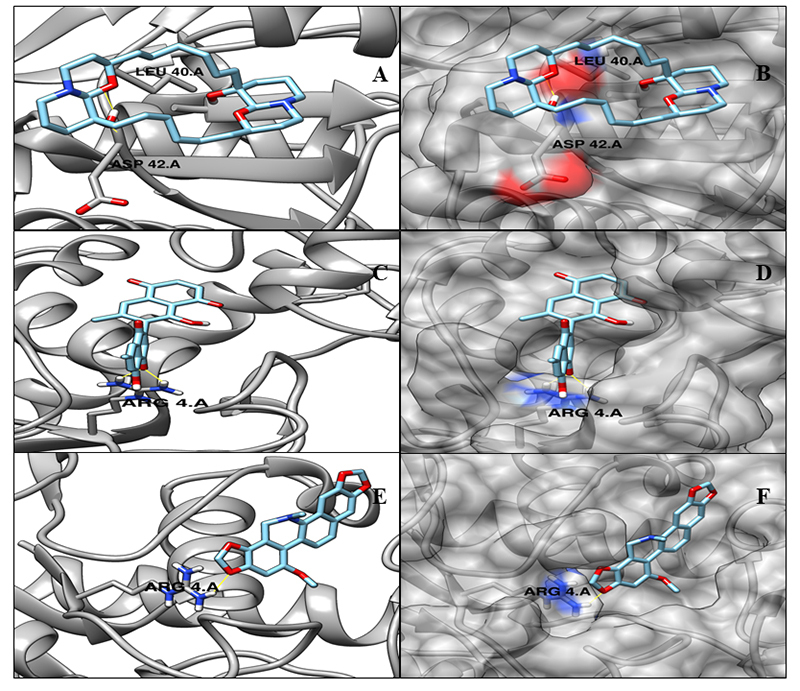
Three-dimensional representation of docking analysis for the target ML0808 (*entC* ML0808). **(A)** Cartoon representation with molecule CID 5276744 [(+)-Araguspongine]. **(B)** Surface representation with molecule CID 5276744 [(+)-Araguspongine]. **(C)** Cartoon representation with molecule CID 308140 (Diospyrin). **(D)** Surface representation with molecule CID 308140 (Diospyrin). **(E)** Cartoon representation with CID 440589 **(**Dihydrochelirubine). **(F)** Surface representation with CID 440589 (Dihydrochelirubine).

Ml_TN_1385 (entC, Putative phosphoglycerate mutase family protein) is involved in the biosynthesis of enterobactin. Concisely, isochorismate is a common predecessor for the siderophore enterobactin and menaquinone (vitamin K2) biosynthesis in *E. coli,* which is shaped by shikimate pathway from chorismate by the enzyme isochorismate synthase encoded by the *entC* gene [54]. A comparison between modeled structure and the template (PDB ID: 2A69) could not identify any active site residue. The docking analysis was performed to identify the high ranked minimum energy binding affinity score. Table 6 and Figure 5 show the set of 3 best interacting ligands according to their minimum affinity and the number of hydrogen bond interactions.

**Table 6.** Autodock vina scores for the selected best-ranked natural compounds with target ML0808 (putative phosphoglycerate mutase family protein) and predicted hydrogen bonds.

Ml_TN_1385 (entCML0808)		
Compound name	Autodock vina binding affinity	N^o^ of H-bond/residues
CID 5276744 [(+)-Araguspongine]	-6.8	3/CYS15, SER11, ALA10
CID 308140 (Diospyrin)	-7.8	2/ARG4
CID 440589 (Dihydrochelirubine)	-7.8	1/ARG4

**Figure 6.**
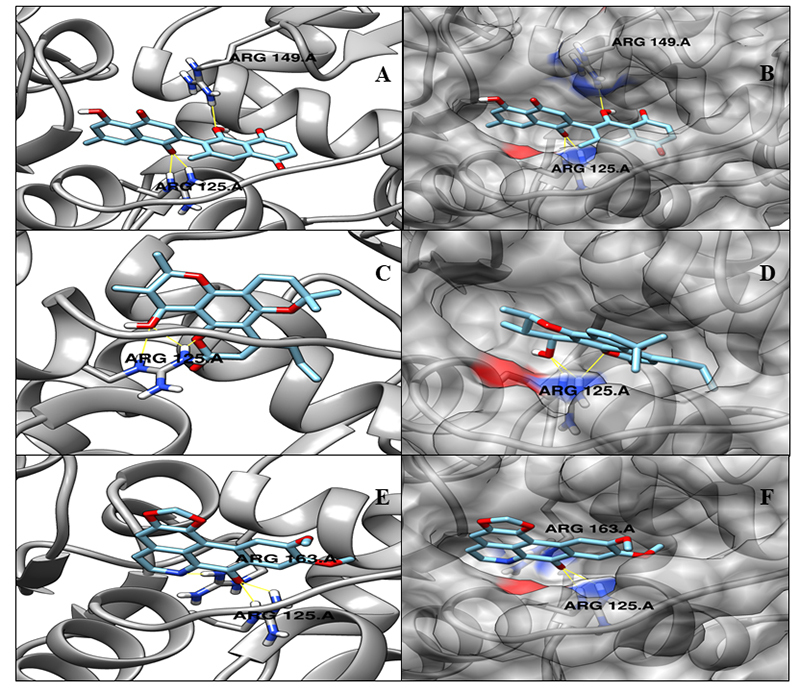
Three-dimensional representation of docking analysis for the target ML2123 (two-component system response regulator). **(A)** Cartoon representation with molecule CID 308140 (Diospyrin). **(B)** Surface representation with molecule CID 308140 (Diospyrin). **(C)** Cartoon representation with molecule CID 64972 (Calanolide A). **(D)** Surface representation with molecule CID 64972 Calanolide (A). **(E)** Surface representation with CID 177744 (Dicentrinone). **(F)** Surface representation with CID 177744 (Dicentrinone).

NP_302402.1 (ML2123, Two-component system response regulator, TCS regulator): the TCS is known as a basic mechanism of stimulus-response coupling that helps in sensing and responding to changes in different environmental conditions in microorganisms. This system is found mostly in bacteria, in domains of microorganisms, and accomplishes signal transduction through phosphorylation of its cognate response regulator. This signaling approach for coupling changes in the environment to cellular physiology is abundant throughout the bacteria. These signaling proteins are found in all the sequenced bacterial genomes [55, 56]. Their abundance differs in each domain, where His-Asp phosphotransfer system accounts for the mainstream signaling pathways in eubacteria, but are rare in eukaryotes [57]. Based on the crystallographic structural comparison with the template (PDB ID: 1YS7), none of the active site residues were identified. The docking analysis was performed to identify the minimum energy binding affinity score. Table 7 and Figure 6 show the set of 3 best interacting ligands according to their minimum affinity and the number of hydrogen bond interactions.

**Table 7.** Autodock vina score for the selected best-ranked natural compounds with target ML2123 (two-component system response regulator, TCS regulator) and predicted hydrogen bonds.

Ml_TN_3807 NP_302402.1 ML2123 - Two-component system response regulator		
Compound name	Autodock vina binding affinity	N^o^ of H-bonds/residues
CID 308140 (Diospyrin)	-9.4	3/ARG125, ARG149
CID 64972 (Calanolide A)	-9.2	4/ARG125
CID 177744 (Dicentrinone)	-8.8	3/ARG125, ARG163

In our docking analysis, the drug molecule Diospyrin (CID 308140) showed good binding affinity with all three drug targets. Diospyrin is a DNA Gyrase inhibitor with a different mechanism of action and it has been reported as a possible therapeutic target against *Mycobacterium tuberculosis* [58]. The binding strength of our identified molecules with Diospyrin suggests that the latter can be potentially used as a new drug for the treatment of leprosy.

## Discussion

Leprosy is an infectious disease that targets skin and peripheral nerves caused by *Mycobacterium leprae* and *Mycobacterium lepromatosis*. It is curable if identified on time, however a delay in diagnosis and treatment, could lead to permanent nerve damage that cannot be retreated by antibiotics. Leprosy is an important health concern around the globe and it is prevalent in many regions of the world and also a public health problem in Brazil. Yearly, more than 30,000 new cases of leprosy are diagnosed in Brazil [59]. Moreover, it presents a wide range of clinical manifestations, which are dependent on pathogen and host interaction, and are allied to the degree of immunity to the bacillus. Currently, the main strategy for the prophylaxis of leprosy is to identify the infection at an early stage and treat it, because there is no specific vaccine against *M. leprae*; although, the BCG vaccine is widely acclaimed and used in endemic countries, with reliable evidence of its protection against leprosy [8, 13] The bacteria have developed resistance against several antibiotics, thereby obliging the scientific community to start investigating new therapeutic targets against *M. leprae* [10, 60]. The comparative genomics, subtractive genomics and reverse vaccinology of 6 genomic strains of *M. leprae* and *M. lepromatosis* identified new vaccine and drug targets that could be tested in the near future in order to solve this public health problem. We identified 12 non-host homologous proteins, which can be used as vaccine candidates and 3 non-host homologous proteins as drug targets. The molecular docking analysis showed Diospyrin (CID 308140) as the most promising compound with the best interactions with our identified drug targets. Compound Diospyrin obtained from indigenous plant Diospyros spp possessing anti-leishmanial [58, 61] was the best drug candidate in our analysis, which has already been reported as a potential therapeutic agent against *Mycobacterium tuberculosis* and could be considered for antimicrobial chemotherapy in future studies for the development of drugs and vaccines against leprosy disease.

## Conclusions

We used bioinformatics approaches in this study for the identification of common potential drug and vaccine candidates against *M. leprae* and *M. lepromatosis*. The 6 genomic strains of *M. leprae* and *M. lepromatosis* were used. Moreover, reverse vaccinology and subtractive genomics approaches were employed for the prediction of new drugs and vaccine candidates. After the detailed *in silico* analysis, we present 12 non-host homologous protein targets as vaccine candidates and 3 non- host homologous proteins as drug targets. We hypothesize that these identified therapeutic targets and antimicrobial drugs [bis-naphthoquinone compound Diospirin (CID 308140)] could be considered for prophylaxis of leprosy and hence should be subjected to further experimental validations.
